# The role of ERK-1 and ERK-2 gene polymorphisms in PCOS pathogenesis

**DOI:** 10.1186/s12958-022-00967-6

**Published:** 2022-06-29

**Authors:** Gurhan Guney, Mine Islimye Taşkın, Nazli Sener, Ezgi Tolu, Yavuz Dodurga, Levent Elmas, Orkun Cetin, Cengiz Sarigul

**Affiliations:** 1 Department of Reproductive Endocrinology and Infertility, Balikesir University Medical Faculty, Cagis Yerleskesi,Bigadic Yolu 17.km, 10145 Balıkesir, Turkey; 2 Department of Medical Biology, Pamukkale University Medical Faculty, Denizli, Turkey; 3 Department of Medical Biology, Bakircay University, Izmir, Turkey; 4 Department of Obstetrics and Gynecology, Balikesir University Medical Faculty, Balıkesir, Turkey; 5 Department of Medical Biochemistry, Ataturk University Medical Faculty, Erzurum, Turkey

**Keywords:** PCOS, ERK-1, ERK-2, Genetic polymorphism

## Abstract

**Background:**

Ovulation is regulated by extracellular signal-regulated kinase-1 (ERK-1) and ERK-2 signaling mechanisms, and ERK-1/2 kinases modulates the function of most of the LH-regulated genes. Defective ERK kinase signaling that is secondary to a genetic problem contributes to both ovulatory dysfunction and metabolic problems in polycystic ovary syndrome (PCOS). We planned to investigate ERK-1 and ERK-2 gene polymorphisms in PCOS for the first time in the Turkish population.

**Methods:**

One hundred two PCOS patients and 102 healthy controls were recruited for this patient control study. HOMA-IR, Ferriman-Gallwey score (FGS), waist-to-hip ratio (WHR), and body mass index (BMI) were assessed. Lipid profile levels, CRP, and total testosterone were determined. ERK-2 rs2276008 (G > C) and ERK-1 rs11865228 (G > A) SNPs were analyzed with a real-time PCR system.

**Results:**

ERK-1 and ERK-2 genotypes were found to differ between the PCOS and control groups. In patients with PCOS, ERK-1 GA and ERK-2 GC genotypes were different in terms of BMI, FGS, HOMA-IR, CRP, total testosterone, and total cholesterol levels.

**Conclusions:**

ERK-1 and ERK-2 genes are involved in PCOS pathogenesis. BMI, FGS, HOMA-IR, and CRP levels are related to the heterozygote polymorphic types of ERK-1 and ERK-2 genes.

## Background

Polycystic ovary syndrome (PCOS) is a common endocrinological and reproductive disorder characterized by hyperandrogenism, polycystic ovaries, and ovulatory dysfunction, which is seen in 6%–10% of the female population [1]. PCOS is also associated with metabolic problems, such as atherosclerosis, insulin resistance, obesity, dyslipidemia, and increased cardiovascular disease. Although the pathogenesis is still unclear, increasing amounts of evidence suggest that genetic factors affect its etiology [2].

Extracellular signal-regulated kinase-1 (ERK-1) and ERK-2 kinases play an important role in transmitting signals from the cell surface into the cell and are involved in many cell processes, including cell adhesion, migration, proliferation, and differentiation and maintenance of the cell cycle [3]. Considerable evidence shows the primary role of the ERK-1/2 signaling cascade in ovulation. Studies have detected that genetic inactivation of the ERK-1 and ERK-2 cascade in mouse granulosa cells causes sterility, along with oocyte meiotic maturation, cumulus expansion, and follicle rupture [4]. Previous microarray analyses disclosed that the destruction of ERK-1/2 changes the structure of approximately 77% of LH-regulated genes and defective ERK kinase signaling that is secondary to a genetic problem contributes to both ovulatory dysfunction and metabolic problems in PCOS. Due to these metabolic problems, anovulation, hyperandrogenemia, and infertility develop [5, 6].

Although it has been shown that there is a relationship between polymorphic variants of some genes and PCOS pathogenesis in various case control studies, most did not obtain a clear result [5, 7]. Furthermore, no existing study has previously evaluated ERK-1 and ERK-2 gene polymorphisms in a Turkish population. We planned to investigate ERK-1 and ERK-2 polymorphisms in our PCOS population, as well as their relation to other metabolic parameters.

## Methods

### Patients

In this case control study, we selected 204 study participants aged 18–35 years from our outpatient gynecology clinic between August 2017 and December 2019. Our research was conducted in accordance with the Helsinki Declaration’s requirements and was approved by the Ethics Committee of the Balıkesir University (number: 2017/48). Patients were divided into two main groups: 102 diagnosed with PCOS and 102 healthy controls without PCOS. Before the study was conducted, informed consent was obtained from all patients. Based on the criteria of the European Society of Human Reproduction and Embryology (ESHRE) and the American Society for Reproductive Medicine (ASRM, 2004) [1], we diagnosed PCOS if two of the following three criteria were present: polycystic appearance of the ovaries on the ultrasound image (the presence of 12 or more follicles from 2–9 mm in diameter and ovarian volume above 10 cm^3^), anovulation and oligo-ovulation, and biochemical and clinical signs of hyperandrogenism. Oligo-anovulation was defined according to the presence of oligomenorrhea (menstrual cycle longer than 35 days) or amenorrhea (failure to menstruate for six months or longer). Blood was drawn in the early follicular period in both patients with menstrual cycles and progesterone-affected patients with amenorrhea. Transvaginal ultrasound was applied on the same day, using a 7.5-MHz vaginal transducer (Voluson 730, GE Healthcare, Austria). A single physician examined all patients. The Ferriman-Gallwey Score (FGS) was used to evaluate hirsutism [8]. Waist to hip ratio (WHR), which indicates visceral fat accumulation and also body mass index (BMI) was calculated. The single physician evaluated the hirsutism scores, WHR, and BMI.

Exclusion criteria were the receipt of treatments including insulin sensitizers, glucocorticoids, anticoagulants, antiandrogens, oral contraceptives, and antiplatelet drugs. In addition to these criteria, patients of advanced age, patients with systemic diseases such as hyperlipidemia, hypertension, and diabetes mellitus, and patients diagnosed with hepatic failure, renal failure, Cushing’s syndrome, hyperprolactinemia, congenital adrenal hyperplasia, and virilizing tumors were also excluded from the study.

### Biochemical analysis

Between 09:00 and 10.00 A.M following an overnight fast, venous blood samples from all patients were sent to the laboratory for hormonal and biochemical analyses. All samples were centrifuged at 825 g for 10 min and stored at − 40 °C until analysis. Commercially available kits (Cobas Integra 800; Roche Diagnostics GmbH, Germany) were used on a chemistry autoanalyzer to measure glucose, low density lipoprotein (LDL), high density lipoprotein (HDL), total cholesterol and triglycerides. In a hormone autoanalyzer, we measured the fasting insulin levels with Access Kits. (Beckman Coulter; Unicel DXI 600; Access Immunoassay, Brea, CA). Serum testosterone levels were measured using commercially available kits (eBioscience, Vienna, Austria) with an enzyme-linked immunosorbent assay (ELISA) technique on a diagnostic instrument (BioTek, ELx 800, Winooski, VT). Insulin resistance was calculated using the homeostasis model (HOMA-IR) as fasting glucose (mmol/l) x fasting insulin (mU/l) / 22.5 [9, 10]. C-Reactive Protein (CRP)concentration was determined by laser nephelometry [11].

### Genomic DNA isolation from venous whole blood samples

In order to isolate genomic DNA from control and PCOS group, we collected 2 ml venous whole blood samples to hemogram tubes (anti-coagulated with K2-EDTA). Before isolation steps, blood samples were gently mixed by vortexing. Genomic DNA of both groups were isolated by QIAamp DNA blood kit (Qiagen, Germany) according to manufacturer’s protocol. Briefly, 200 µl whole blood samples, 20 µl proteinase K and 200 µl AL Buffer were added into 1.5 ml microcentrifuge tube and mixed by vortexing for 15 s. The mixture were incubated at 56^O^C for 10 min. Following incubation step, 200 µl ethanol (96–100%) were added to the sample. Then, total mixture were transferred to QIAamp Mini spin column (in a 2 ml collection tube) and centrifugated at 8000 rpm for 1 min. After centrifugation step, columns were washed with 500 µl AW1 buffer and centrifugated at 8000 rpm for 1 min. Subsequent to AW1 buffer, columns were washed with AW2 buffer and centrifugated at 14,000 rpm for 3 min. Finally, columns were placed to clean 1.5 ml microcentrifuge tube and 200 µl Buffer AE were added. After the centrifugation step at 8000 rpm for 1 min, we obtained DNA. The concentration and quality of DNA samples were measured spectrophotometrically via NanoDrop (Thermo, USA). DNA samples were stored -20^O^C until genotyping analysis.

### SNP Genotyping of *MAPK1* (*ERK2*) and *MAPK3* (*ERK1*) Genes

As a result of the NCBI SNP screening (https://www.ncbi.nlm.nih.gov/snp/), a total of 23dbSNP and 263 dbvar polymorphic regions were detected for the MAPK1 gene. Likewise, a total of 16 dbSNPs and 593 dbvar polymorphic regions were detected for the MAPK3 gene. For our study, rs2276008 and rs11865228 polymorphisms, which have not been studied with PCOS, were selected among 23 SNPs for MAPK1 and for MAPK3 from 16 SNPs, respectively, which we think will contribute more to the literature among these SNPs. In order to analyse *MAPK1* (*ERK2*) rs2276008 (G > C) and *MAPK3* (*ERK1*) rs11865228 (G > A) single nucleotide polymorphisms, we used Taqman based SNP assays (Thermo, USA) and genotyping analysis was performed via StepOne Plus real time PCR system (Thermo, USA). We prepared 20 µl reaction mix for both SNPs as following: 12.5 µl Taqman Universal PCR Master Mix (2X), 1.25 µl Taqman Genotyping Assay Mix (20X), 6.25 µl DNase-free water. 5 µl DNA for each sample. After mixture preparation, 20 µl total mixture were transferred to StepOne Plus 96-multiwell plate and 5 µl sample DNA were added tor related wells. The plate sealed with its optical adhesive film before running the experiment. StepOne Plus instrument method was performed as following: denaturation for 10 min at 95^O^C; following 40 cycles including denaturation for 15 s at 95^O^C, annealing for 1 min at 60^O^C.

### Statistical analysis

The Statistical Package for the Social Sciences (SPSS) statistical program (SPSS, version 25.0, Chicago, IL) was used in all statistical analyses. The Kolmogorov–Smirnov test was used to determine whether the distributions of continuous variables were normal. Data were described as mean ± SD. For variables that did not show a normal distribution, we used the Mann–Whitney U test to compare the two groups. For normally distributed parameters, we used a T-test to determine the differences between the two groups. In order to compare multiple variables,one-way ANOVA was used to determine differences among the groups. The homogeneity of variances was tested with the Levene test. When the p-value from one-way ANOVA test statistics was statistically significant, the post hoc Tamhane test was used. Because variances were not homogenic and the number of subgroups was different, the correlations between the data were evaluated by Spearman’s rank correlation analyses. Differences were considered statistically significant with a *p*-value < 0.05.

## Results

Clinical, biochemical, and hormonal parameters of the patients are shown in Table 1. Age and HDL levels were similar between the PCOS and control groups (*p* > 0.05). HOMA-IR, FGS, BMI, CRP, total testosterone, LH/FSH ratio, triglycerides, LDL, and total cholesterol levels were higher in the PCOS group than the control group (*p* < 0.05).

**Table 1:** Clinical characteristics, hormonal and biochemical results of the study groups

Variables	PCOS (*n* = 102)	Control (*n* = 102)	*p-*value
Age (years)	30.88 ± 8.36	30.68 ± 4,36	0.07
BMI (kg/m^2^)	29.02 ± 5.64	26.55 ± 6.07	***p* ≤ 0.001**
WHR	0.78 ± 0.22	0,71 ± 0.14	0.006
Ferriman Gallwey Score (FGS)	12.43 ± 5.80	5.88 ± 2.06	***p* ≤ 0.001**
HOMA-IR	2.92 ± 1.89	1.78 ± 0.97	***p* ≤ 0.001**
CRP	11.67 ± 2.73	6.53 ± 3.85	***p* ≤ 0.001**
LH/FSH ratio	1.30 ± 1.03	0.78 ± 0.56	***p* ≤ 0.001**
Total Testosterone (ng/dl)	0.74 ± 0.36	0.51 ± 0.20	***p* ≤ 0.001**
Triglycerides (mg/dl)	117.85 ± 48.89	91.80 ± 38.09	***p* ≤ 0.001**
Total Cholesterol (mg/dl)	192.21 ± 43.59	169.45 ± 30.12	***p* ≤ 0.001**
LDL (mg/dl)	116.95 ± 41.63	99.50 ± 26.46	***p* ≤ 0.001**
HDL (mg/dl)	51.70 ± 10.45	52.74 ± 11.54	0.848

Data presented as Mean ± SD

*P* < 0.05 accepted as  statistically significant

Distributions of the genotypes in ERK-1 and ERK-2 genes are shown in Table 2. ERK-1 and ERK-2 genotypes differed between the PCOS and control groups. The GA genotype in ERK-1 and the GC genotype in ERK-2 were higher in patients with PCOS than in the control group. The GG genotype was higher in the control group than in the PCOS group in both ERK-1 and ERK-2 genes (*p* = 0.001). Multiple comparisons of ERK-1 genotypes and clinical, biochemical and hormonal parameters are given in Table 3. All parameters except HDL differed significantly between the groups. BMI and FGS were higher in P(PCOS)-GA than in P-GG. HOMA-IR and total testosterone were higher in P-GA than in C(Control)-GA. BMI, FGS, HOMA-IR, CRP, total testosterone, and total cholesterol were higher in the P-GA than the C-GG genotype. Except BMI and HDL, all parameters differed between P-GG and C-GG. FGS, CRP, total testosterone, and triglycerides were higher in the P-GG group than in the C-GA group.

**Table 2:** ERK 1 and ERK 2 genotypes distrubutions in PCOS versus control group

	**PCOS Group**	**Control Group**
	***n* = 102**	***n* = 102**
**ERK-1 Genotypes**		
GG	50 (% 49.0)	82 (% 80.4)
GA	52 (% 51.0)	20 (%19.6)
***p* value**	***p* ≤ 0.001**	
**ERK-2 Genotypes**		
GG	42 (% 41.2)	80 (%78.4)
GC	60 (% 58.8)	22 (% 21.6)
***p* value**	***p* ≤ 0.001**	

*P* < 0.05 accepted as  statistically significant

**Table 3:** Comparisons between ERK 1 genotypes and clinical characteristics, biochemical-hormonal parameters

	**PCOS-ERK 1**		**Control-ERK 1**		***P***^**a**^
**Parameters**	P-GG	P-GA	C-GG	C-GA	
BMI	27,3 ± 5,06^b^	30,68 ± 5,72^c^	26,11 ± 5,41	28,38 ± 8,15	**≤ 0.001**
FGS	11,08 ± 4,01^b,f^	13,73 ± 6,9^c^	5,48 ± 1,84	7,5 ± 2,16^e^	**≤ 0.001**
HOMA-IR	2,70 ± 2,12^f^	3,13 ± 1,63^c,d^	1,75 ± 0,97	1,89 ± 0,96	**≤ 0.001**
CRP	11,38 ± 3,05^f^	11,96 ± 2,38^c^	6,40 ± 4,06	7,06 ± 2,86^e^	**≤ 0.001**
Total testesteron	0,71 ± 0,33^f^	0,77 ± 0,39^c,d^	0,53 ± 0,19	0,4 ± 0,21^e^	**≤ 0.001**
Triglyceride	125,54 ± 50,53^f^	110,46 ± 46,54	93,43 ± 39,7	85,1 ± 30,46^e^	**≤ 0.001**
Total Cholesterol	196,52 ± 41,65^f^	188,07 ± 45,39^c^	168,9 ± 26,18	171,7 ± 43,5	**≤ 0.001**
LDL	116,89 ± 38,3^f^	117,01 ± 44,92	99,28 ± 21,4	100,4 ± 42,05	**0.006**
HDL	54,56 ± 10,77	48,96 ± 9,44	52,53 ± 12,75	53,6 ± 3,87	**0.64**

Data presented as Mean ± SD and *p* < 0.05 accepted as statistically significant

^a^ Groups were compared between P-GG,P-GA,C-GG and C-GA with; one-way ANOVA test

^b ^*p* < 0.05 (P-GG vs P-GA); Tamhane test

^c ^*p* < 0.05 (P-GA vs C-GG); Tamhane test

^d ^*p* < 0.05 (P-GA vs C-GA); Tamhane test

^e ^*p* < 0.05 (P-GG vs C-GA); Tamhane test

^f ^*p* < 0.05 (P-GG vs C-GG); Tamhane test

P: PCOS (Polycystic Ovary Sydrome) Group

C: Control Group

In Table 4, comparisons between clinical characteristics and biochemical–hormonal parameters in ERK-2 genotypes are given. BMI, FGS, and HOMA-IR were significantly higher in P-GC than in P-GG. All parameters except HDL were higher in P-GC than in C-GG. FGS, CRP, total testosterone, LDL, total cholesterol, and triglycerides were different in P-GG and C-GG. Only triglycerides were higher in P-GG than in C-GC. BMI, FGS, HOMA-IR, and triglycerides were higher and HDL was lower in the P-GC than in the C-GC genotype. Correlation analyses of all parameters showed that CRP positively correlated with BMI, HOMA-IR, and total testosterone. BMI was also positively correlated with HOMA-IR and total testosterone**.**

**Table 4:** Comparisons between ERK 2 genotypes and clinical characteristics, biochemical-hormonal parameters

	**PCOS-ERK 2**		**Control-ERK 2**		***P***^***a***^
**Parameters**	P-GG	P-GC	C-GG	C-GC	
BMI	25,49 ± 3,88^b^	31,5 ± 5,38^c,d^	26,3 ± 6,25	27,29 ± 5,43	**≤ 0.001**
FGS	9,76 ± 3,04^b,f^	14,3 ± 6,52^c,d^	5,20 ± 1,67	8,36 ± 1,32	**≤ 0.001**
HOMA-IR	2,24 ± 1,55^b^	3,40 ± 1,97 ^c,d^	1,76 ± 0,79	1,84 ± 1,47	**≤ 0.001**
CRP	11,16 ± 2,59^f^	12,03 ± 2,80^c^	5,39 ± 2,24^g^	10,67 ± 5,45	**≤ 0.001**
Total testesteron	0,72 ± 0,36^f^	0,75 ± 0,37^c^	0,48 ± 0,2	0,6 ± 0,18	**≤ 0.001**
Triglyceride	122 ± 53^e,f^	114,95 ± 46,03^c,d^	93,17 ± 40,87	86,81 ± 25,7	**≤ 0.001**
Total Cholesterol	191,52 ± 41,98^f^	192,7 ± 45,03^c^	164,87 ± 27	186,09 ± 35,4	**≤ 0.001**
LDL	113,31 ± 37,75	119,5 ± 44,28^c^	96,26 ± 23,8	111,27 ± 32,43	**≤ 0.001**
HDL	53,80 ± 10,51	50,23 ± 10,24^d^	51,12 ± 11,12	58,63 ± 11,39	**≤ 0.001**

Data presented as Mean ± SD and *p* < 0.05 accepted as statistically significant

^a^ Groups were compared between P-GG,P-GC,C-GG and C-GC with; one-way ANOVA test

^b ^*p* < 0.05 (P-GG vs P-GC); Tamhane test

^c ^*p* < 0.05 (P-GC vs C-GG); Tamhane test

^d ^*p* < 0.05 (P-GC vs C-GC); Tamhane test

^e ^*p* < 0.05 (P-GG vs C-GC); Tamhane test

^f ^*p* < 0.05 (P-GG vs C-GG); Tamhane test

^g ^*p* < 0.05 (C-GG vs C-GC); Tamhane test

P: PCOS (Polycystic Ovary Sydrome) Group

C: Control Group

## Discussion

This study has demonstrated that one of the most significant and largest extracellular and intracellular signaling systems, called ERK-1 and ERK-2 kinases, plays a role in PCOS pathogenesis. We observed that heterozygote genotypes of ERK-1 and 2 genes were significantly higher in PCOS and that these polymorphic types were related to some clinical, biochemical and hormonal parameters, such as BMI, FGS, HOMA-IR, and CRP levels. According to the Reference SNP Report [12], the incidence of GA of ERK-1 is generally 1.2–3.4% and the incidence of GC of ERK-2 is 7–8%, or 15–18% in Korea. In our study, the incidences of GA of ERK-1 and GC of ERK-2 were 19.6% and 21.6%, respectively [13]. The incidence rates found in our study, which are high compared to other populations, may be unique to Turkey, as our study is the first study to conduct SNP analyses of ERK-1 and ERK-2 genes in the Turkish population. Comprehensive studies conducted by Rivas et al. [12] and Ellegren et al. [14] found results showing that the same genes have different frequencies of polymorphism in different populations, which might explain this high rate of difference.

In one *in vivo* study, it was shown that LH hormone needed ERK-1 and ERK-2 pathways in order to initiate meiosis and to continue the ovulation and luteinization phases in granulosa cell culture, sequentially and without error [15]. In another study, it was observed that due to a mutation in ERK-1/2 kinase genes, oocyte maturation was impaired by a complete lack of germinal vesicle breakdown with impaired coronocumulus cell expansion and corpus luteum formation due to a lack of follicle rupture [16]. This problem of cell expansion and follicle rupture may be due to anovulation, which partially explains the excessive number of follicles accumulated in the ovaries [17, 18].

In addition to anovulation, increased intraovarian testosterone levels contribute to the clinical picture of PCOS. Nelson-Degrave et al. [19] and Corbould et al. [20] claimed that excessive ovarian androgen production in women with PCOS was secondary to abnormal activation of the ERK signaling pathway. Other publications assert the opposite. For example, in a study of endometrial cancer patients previously diagnosed with PCOS, Lin et al. [21] concluded that higher testosterone levels cause the phosphorylation of the ERK pathway, which results in an increase in its activity. In addition to this phosphorylation, they also observed that testosterone has a carcinogenic effect by. transforming into estrogen with aromatase enzyme after binding to ER-α36, which activates the ERK pathway As aromatase enzyme activity varies from person to person, and as Lin et al.`s study was conducted *in vitro*, the results are expected to be confirmed by a large number of studies [22]. In our study, testosterone levels were significantly higher in the PCOS group, especially in the GC genotype for ERK-2 and the GA genotype for ERK-1. Similar to our study, research by Hu et al. [23] on Chinese women showed that polymorphic variants of ERK-1 and ERK-2 increased the risk of PCOS development. In the same study, they detected that MAPK signaling was activated by the LH receptor and serum testosterone levels were increased if the MAPK kinase inhibitor PD98059 was given to those patients.

Various studies of PCOS demonstrated that an intersection between reproductive dysfunction and metabolic problems indicates the possible role of adipose tissue dysfunction in PCOS pathogenesis [24, 25]. Hence, Manneras-Holm et al. ( [26] observed aberrant adipose tissue morphology and function in both underweight and overweight patients with PCOS. To better reveal the role of adipose tissue in PCOS pathogenesis, Kokosar et al. [27] investigated whether there was a genetic change in adipose tissue and found a difference in the activity of many gene pathways in PCOS patients, including in the ERK/MAPK signaling system. Although the number of control patients in that study was small, the screening of a large gene family in patients with PCOS suggests that the results are reliable. In our study, we did not directly evaluate adipose tissue but believed that ERK/MAPK kinase polymorphism affected many tissues in the body, including adipose tissue. Thus, in our study, PCOS patients had higher total cholesterol, LDL, and triglyceride levels compared with the control group. We found that the P-GC genotype for ERK-2 had higher total cholesterol, LDL, and triglyceride levels than the C-GG genotype. This irregularity in lipid levels may be related to different ERK-2 gene polymorphisms and increased lipolysis in patients with PCOS.

Many studies in the literature conclude that there is a relationship between PCOS and chronic low-grade inflammation. Although these studies indicate that this relationship is due to increased BMI, insulin resistance, or hyperandrogenemia accompanying PCOS, no clear reason has been revealed [28]. In our study, we detected significantly higher CRP levels in the PCOS group compared to the control group, and these CRP levels were positively correlated with total testosterone, HOMA-IR, and BMI levels. We also wished to examine whether there is a correlation between ERK-1 and ERK-2 kinase polymorphic types and other metabolic parameters, and we detected a significant difference between BMI, HOMA-IR, and CRP levels with both ERK heterozygote types. Based on these results, we may say that there is a sensitive and complex relationship between ERK pathways and inflammation, insulin resistance, and hyperandrogenemia.

Like most studies in the literature, ours has limitations and strengths. As the first study in our population to investigate the ERK-1 and ERK-2 polymorphisms and their relationship with other metabolic parameters in patients with PCOS, novelty is the strongest part of our study. The limitations of the study are that we did not evaluate infertility in patients with PCOS, nor did we classify our patients into obese and lean PCOS groups according to BMI. It is known that the SNP of ERK-1/2 on the 3'-UTR does not cause loss of mRNA`s function, as ERK-1/2 on the 3'-UTR is not found in the open reading frame. Although this may first appear to be a limitation, 3'-UTR mutations affect the half-life of the mRNA by causing mRNA instability, which may ultimately reduce both the function and amount of the end protein product. The presence of fewer ERK-1 and ERK-2 kinase protein in the cell will cause irregularity in the metabolic pathways they control.

## Conclusions

PCOS is a complex disease characterized by metabolic disorder and infertility. Its association with anovulation, insulin resistance, hyperandrogenism, and inflammation is attributable to ERK-1 and ERK-2, the largest signalization system to manage major vital processes, such as cellular proliferation and differentiation. In this study, we showed that polymorphism in MAPK/ERK genes is related to PCOS risk in Turkish women. In order to understand whether SNP mutations alter ERK signaling, we believe that performing depletion and alteration studies on cell lines such as KGN cells using expression vectors encoding these mutations can greatly increase the value of our study.

## Data Availability

The datasets used and/or analysed during the current study are available from the corresponding author on reasonable request.

## Abbreviations

ERK-1: Extracellular signal-regulated kinase-1
ERK-2: Extracellular signal-regulated kinase-2
HOMA-IR: Homeostatic Model Assessment for Insulin Resistance
FGS: Ferriman-Gallwey score
WHR: Waist-to-hip ratio
BMI: Body mass index
CRP: C-Reactive Protein
SNPs: Single nucleotide polymorphisms
LH: Luteinizing hormone
